# Supplementary material for: The genome sequence of a cranefly, Tipula ( Lunatipula) helvola Loew, 1873
Source: Wellcome Open Res. 2024 Oct 16;9:597. [Version 1] doi: 10.12688/wellcomeopenres.23207.1 (PMC12405859; doi:10.12688/wellcomeopenres.23207.1)
Supplement: Peer review history

# Reviewer report: Reviewer response for version 1

**DOI:** 10.21956/wellcomeopenres.25559.r129588

**Published:** 2025-09-02

**Authors:** Chelsea Skojec

**Affiliations:** 1 University of Florida, Gainesville, Florida, USA

This is a clear and robust Genome Note reporting a high quality genome assembly for *Tipula helvola*. The rationale is sound, a reference genome for a nationally scarce cranefly will support taxonomy, phylogeny, and biology. The sequencing and assembly strategy uses PacBio HiFi with Hi C scaffolding, with Hifiasm followed by purge_dups and then YaHS. Reported quality metrics are strong for a non model Dipteran, with a QV about 56, k mer completeness about 99.99 percent, and BUSCO completeness about 94.6 percent based on the Diptera odb10 reference set. Data and methods are well documented and linked to open repositories and protocols.

 Minor Suggestions:

 In Figure 3, “Blot plot” looks like a typo. Use “Blob plot.”

 In the Hi-C fixation description, state the final formaldehyde percentage used during fixation, since 22 percent likely refers to the stock.

 Make the chromosome count consistent. The text says four chromosomal pseudomolecules and 96.43 percent assigned to them, while the table snippet shows three. Ensure all four are listed with names and lengths to match the Hi-C map.

 Clarify the sex chromosome sentence. For example, “No half-coverage chromosome was observed in the female sample, consistent with XX coverage patterns.”Consider adding fragmented and missing BUSCO counts, along with GC percentage and a brief repeat content summary in the main text or in Table 2.

 Add explicit run accessions for the PacBio HiFi and Hi-C libraries in Table 1 to improve findability.

Are sufficient details of methods and materials provided to allow replication by others?

Yes

Is the rationale for creating the dataset(s) clearly described?

Yes

Are the datasets clearly presented in a useable and accessible format?

Yes

Are the protocols appropriate and is the work technically sound?

Yes

Reviewer Expertise:

Evolution of anti-predator traits and mimicry, distribution modeling, comparative genomics in insects, especially Lepidoptera.

I confirm that I have read this submission and believe that I have an appropriate level of expertise to confirm that it is of an acceptable scientific standard.

# Reviewer report: Reviewer response for version 1

**DOI:** 10.21956/wellcomeopenres.25559.r129581

**Published:** 2025-09-02

**Authors:** Edson Ishengoma

**Affiliations:** 1 University of Dar es Salaam, Dar es Salaam, Tanzania

This data note presents the high-quality chromosomal genome assembly of the cranefly *Tipula (Lunatipula) helvola*, a species of conservation interest in Britain. Beyond its immediate taxonomic value, this resource has significant broader implications.

 Scientifically, it provides a crucial new data point for comparative genomics and evolutionary studies within Diptera (true flies), a hyper-diverse order that includes major disease vectors, agricultural pests, and model organisms. This genome will help refine phylogenetic trees, study chromosome evolution, and investigate the genetic basis of adaptations to specific habitats like dry woodlands.

 Economically and agriculturally, while *T. helvola* itself is not a known pest, its genome serves as a key reference for understanding related crane fly species (e.g., *Tipula paludosa*) that are severe agricultural pests, damaging pasture and cereal crops. Insights into their genetics could reveal novel targets for more specific and sustainable biocontrol methods, potentially reducing reliance on broad-spectrum pesticides.

 From a conservation perspective, the genome is a vital tool for monitoring the genetic health of *T. helvola* populations, understanding their recent range expansion in response to environmental change, and informing evidence-based conservation strategies.

 Finally, this work is a product of the landmark Darwin Tree of Life Project, demonstrating the feasibility and value of sequencing all known eukaryotic species in a region. It contributes to a transformative biological resource that will accelerate discovery across the life sciences for decades to come, fostering open science and global collaboration.

Are sufficient details of methods and materials provided to allow replication by others?

Yes

Is the rationale for creating the dataset(s) clearly described?

Yes

Are the datasets clearly presented in a useable and accessible format?

Yes

Are the protocols appropriate and is the work technically sound?

Yes

Reviewer Expertise:

Evolutionary Genomics; Bioinformatics

I confirm that I have read this submission and believe that I have an appropriate level of expertise to confirm that it is of an acceptable scientific standard.
